# Survival and prognostic factors in patients with oral squamous cell carcinoma

**DOI:** 10.4317/medoral.24242

**Published:** 2020-10-09

**Authors:** Andressa Kelly Alves Ferreira, Sérgio Henrique Gonçalves de Carvalho, Ana Flávia Granville-Garcia, Dmitry José de Santana Sarmento, Gustavo Gomes Agripino, Mauro Henrique Nogueira Guimarães de Abreu, Maria Cecília Freire de Melo, Arnaldo de França Caldas Jr, Gustavo Pina Godoy

**Affiliations:** 1 Dentistry postgraduate program, Universidade Federal de Pernambuco (UFPE), Recife, Pernambuco, Brazil; 2 Department of Dentistry, Universidade Estadual da Paraíba (UEPB), Araruna, Paraíba, Brazil; 3 Dentistry postgraduate program, Universidade Estadual da Paraíba (UEPB), Campina Grande, Paraíba, Brazil; 4 Department of Community and Preventive Dentistry, Universidade Federal de Minas Gerais (UFMG), Belo Horizonte, Minas Gerais, Brazil; 5 Department of Pathology, Universidade Federal de Pernambuco (UFPE), Recife, Pernambuco, Brazil

## Abstract

**Background:**

This study aimed to evaluate sociodemographic and clinical factors influencing overall survival (OS) in patients with oral squamous cell carcinoma (OSCC).

**Material and Methods:**

Medical charts of 547 patients with OSCC from a public hospital in northeastern Brazil seen between 1999 and 2013 were evaluated. Survival analysis was performed using the Kaplan-Meier method. The influence of age, sex, ethnicity, clinical stage, anatomical location, type of treatment, and comorbidities on the patients’ prognosis was evaluated. Cox proportional hazards regression model was used to identify independent prognostic factors.

**Results:**

The 5-year OS was 39%. Multivariate analysis showed that age < 40 years (HR = 2.20; 95%CI: 1.02-4.72) and a single treatment modality (HR = 1.91; 95%CI: 1.37-2.67) were associated with a poor prognosis, while early clinical stage resulted in better outcomes (HR = 0.38; 95%CI: 0.25-0.58).

**Conclusions:**

OSCC patients in advanced clinical stages, diagnosed at a younger age, and submitted to a single therapeutic modality have a poorer prognosis.

** Key words:**Head and neck cancer, oral cancer, oral squamous cell carcinoma, survival, prognosis.

## Introduction

Oral squamous cell carcinoma (OSCC) is the most common neoplasm of the oral cavity, which accounts for about 95% of all cases of head and neck cancers. This neoplasm has a multifactorial etiology and commonly affects men between the sixth and seventh decades of life with smoking and drinking habits (1,2).

An increase in oral cavity cancer cases is observed in Brazil. Recent reports estimate that approximately 23,000 new oral cavity and oropharyngeal cancer cases are expected for each year of the 2020-2022 triennium (3).

Despite the advances made in diagnosis and treatment, the prognosis of OSCC continues to be unfavorable, with low 5-year survival rates being reported in the literature. OSCC thus represents an important public health problem. In 2018, about 177,000 deaths related to lip and oral cavity cancer occurred worldwide (4).

Several variables have been pointed out as prognostic factors in individuals with OSCC such as age, gender, and socioeconomic factors, as well as clinical and pathological features of the tumor, including anatomical location, histopathological grading, and treatment modality. However, results regarding the association between survival time and prognostic and predictive factors remain unclear (5,6).

Considering the importance of providing updated information about predictive factors and prognosis of OSCC for health planning and management, this study aims to estimate the survival of patients with OSCC and to evaluate the influence of sociodemographic and clinical features on patient survival time.

## Material and Methods

- Ethical aspects

This study was conducted in full accordance with the World Medical Association’s Declaration of Helsinki as revised in 2013 and received approval from the Human Research Ethics Committee of the State University of Paraíba (Brazil).

- Sample and data collection

This study included data obtained from the medical charts of 547 patients with pathologically confirmed OSCC who were treated from 1999 to 2013 at an oncology referral center in the state of Paraíba, northeastern Brazil. The patient charts were reviewed to identify the sociodemographic profile, tumor characteristics, and proposed treatment.

- Variables

The following sociodemographic variables were obtained: gender, ethnicity (white or non-white), age (≤ 40 and > 40 years), habits of smoking and/or alcohol consumption, and the existence of systemic and/or oral comorbidities. Systemic comorbidities included hypertension, diabetes, depression, sexually transmitted infections, and hematological, hepatic, pulmonary, gastrointestinal, renal and cardiac pathologies. Oral comorbidities comprised xerostomia, mucositis, limited mouth opening, dysphonia, dysgeusia, dysphagia, and candidiasis. This information was assessed in multiprofessional records.

The anatomical site of the tumor was categorized according to ICD-10 (7) into lip (C00-0 to C00-2) and oral cavity (C00-3 to C10, except 07-08). The stage of disease was determined according to the American Joint Committee on Cancer (AJCC) staging criteria (7th Cancer Staging Manual), corresponding to early (I and II) and advanced stages (III and IV) (8).

Treatment was classified as single therapy when surgery, radiotherapy or chemotherapy was applied alone, or as combination therapy when one therapy was used in combination with one or more of the aforementioned modalities.

Overall survival (OS) was calculated from the date of histopathological diagnosis of primary OSCC to the date of death or last follow-up.

- Statistical analysis

Descriptive analysis was performed for sample characterization. The probability of survival was estimated using the Kaplan-Meier method. Cox models were used to estimate the unadjusted and adjusted hazard ratio (HR, 95% CI). Each covariate was included separately in the Cox model and the unadjusted HR (95% CI) was estimated. Covariates with a *p-value* of less than 0.25 were included in the final Cox model and only variables with a *p-value* less than 0.05 remained. Graphical analysis was used to evaluate the adequacy of the variables for the assumption of proportional risks. All statistical analyses were performed using the SPSS software for Windows, version 23.0 (SPSS Inc., Chicago, IL, USA).

## Results

A total of 547 patients were diagnosed with primary OSCC between 1999-2013 at the reported cancer center. The patient characteristics are summarized in Table 1. Most patients were male (63.7%), older than 40 years (97.4%), and white (61.1%). Systemic and oral comorbidities were found in only 36.6% and 27.8% of the patients, respectively. A high percentage of patients (74.4%) had a history of smoking and/or alcohol consumption.

The most common anatomical site of OSCC was the oral cavity (88.1%) and most tumors were diagnosed in advanced stages (54.8%). Regarding treatment modality, combination therapies were the most common in the sample (56.7%).

**Figure 1:**
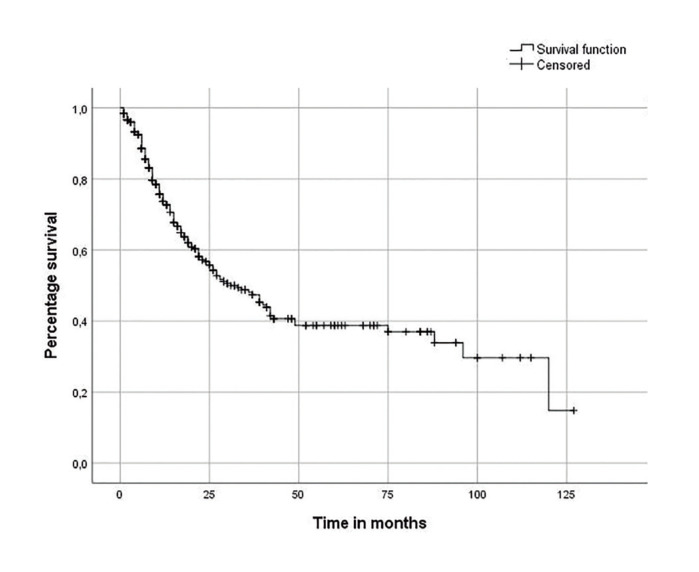
Kaplan-Meier overall survival curve for patients with oral squamous cell carcinoma (n=547) (Recife, 2020).

The 5-year OS of this sample was 39% and the mean and median time of cancer survival was 56 and 31 months, respectively. Fig. 1 shows the Kaplan-Meier survival curve of the patients evaluated.

**Table 1:**
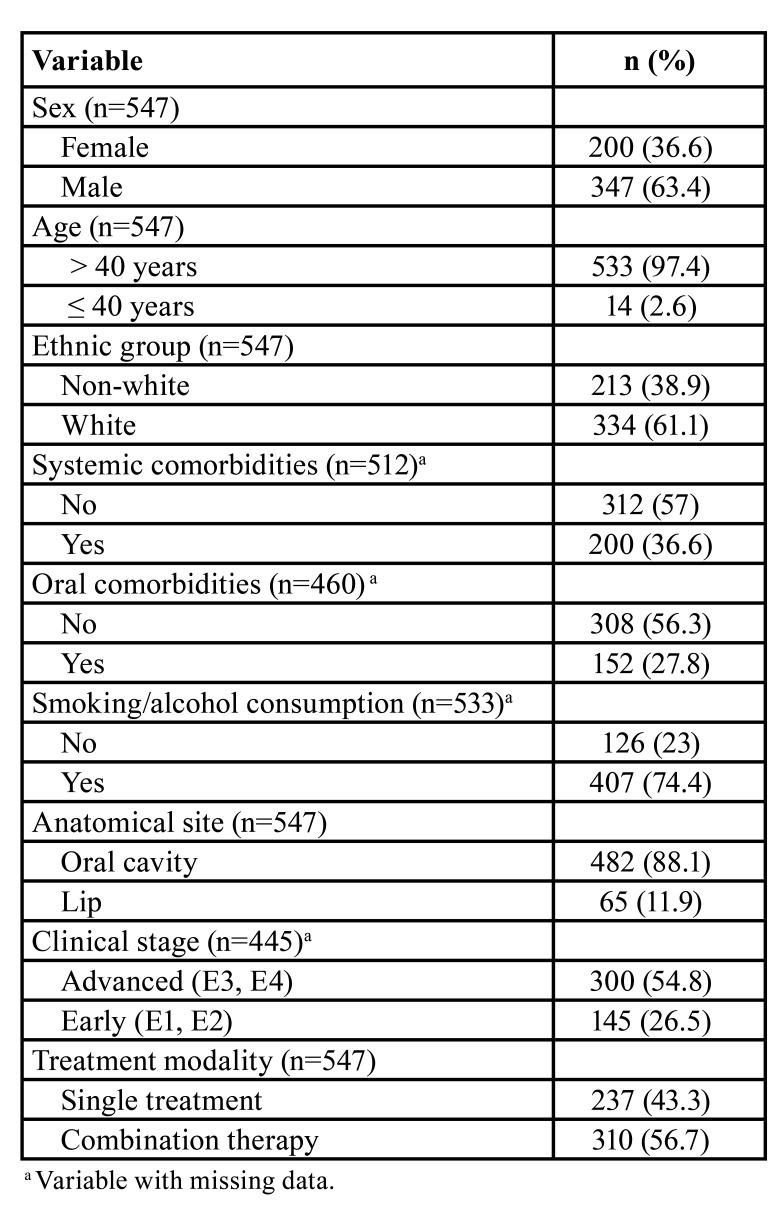
Sample characterization (Recife, 2020).

Multivariate analysis revealed that, among the variables studied, age (*p* = 0.044), clinical stage (*p* < 0.001), and treatment (*p* < 0.001) were covariates associated with survival time (Table 2). The results show that individuals aged up to 40 years had a more than two-fold higher risk of death than older patients (HR = 2.20; 95%CI 1.02-4.72). Patients in early clinical stages had a lower risk of death compared to advanced stages (HR = 0.38; 95%CI 0.25-0.58). The type of treatment also influences the risk of death. Patients treated with a single therapeutic approach were almost twice as likely to die from OSCC (HR = 1.91; 95%CI 1.37-2.67).

**Table 2:**
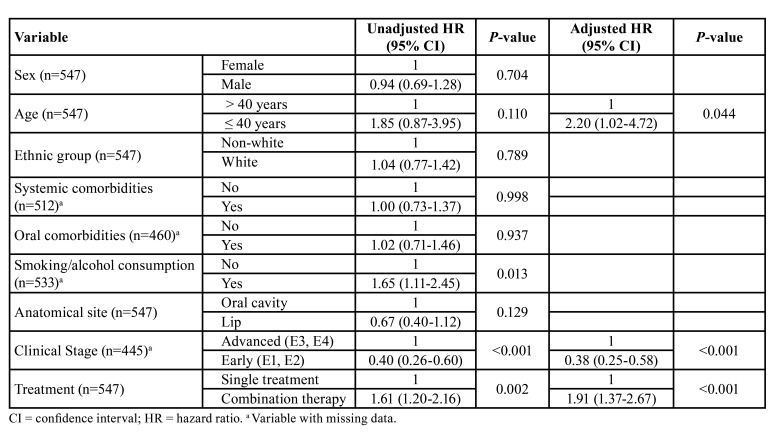
Multivariate analysis of risk factors for overall survival of patients with oral squamous cell carcinoma using Cox regression (Recife, 2020).

## Discussion

The 5-year OS rate of the current study can be considered low when compared to patients with OSCC in other reports (6,9). Furthermore, the findings showed that age ≤ 40 years and a single treatment modality were predictors of poorer overall survival, while early clinical stages provided better outcomes in OSCC patients.

Regarding age, despite the low prevalence of young patients in the sample, the findings showed a higher mortality risk among younger individuals, in agreement with earlier studies (10,11). One factor linked to poorer outcomes in younger patients is a different and more aggressive behavior of the lesions, which could be associated with genetic alterations (12,13). Younger patients are also more likely to have lymphatic metastasis at the time of diagnosis and to have poorly differentiated OSCC, which may result in treatment failure and higher recurrence rates (14,15). Furthermore, OSCC in younger individuals is commonly found at the base of the tongue and oropharynx, which are subsites frequently associated with a poor prognosis (10,14). In contrast, other authors found poorer survival in older individuals. This fact has been associated with comorbidities, which usually limit treatment choices and enhance the likelihood of complications (16,17). Evidence also highlights the increasing association between HPV infection and oropharyngeal lesions in younger patients, with HPV-positive cancers presenting a better treatment response and survival than HPV-unrelated tumors (18).

In the present study, no statistically significant associations were found between survival rates and gender or ethnicity. Other studies identified a poorer prognosis of OSCC in male individuals compared to females (09,16). The higher prevalence and mortality from OSCC in men are usually linked to their increased exposure to risk factors such as tobacco and alcohol consumption. Furthermore, the frequency of health-seeking behaviors tends to be lower in male patients, a fact that can delay the diagnosis and treatment initiation (19). Regarding ethnicity, studies found no correlation between ethnic/racial characteristics and survival in OSCC when adjusted for parameters such as tumor characteristics and treatment (20,21). However, some evidence indicates that ethnicity may play an important role in OSCC survival, with the observation of lower survival rates among black individuals (22). This poorer prognosis has been attributed to lower socioeconomic status and educational levels among blacks when compared to whites, which tend to highly expose this group to more risk factors and hinder access to health services (23). Moreover, black patients had an increased risk of presenting locally advanced and untreaTable oral tumors (24).

Cox regression analysis indicated that advanced clinical stages were associated with poorer survival in OSCC patients. This result is compatible with other studies showing that patients with OSCC are commonly diagnosed late, a fact that negatively influences their prognosis (16,25). This late diagnosis has been attributed to factors such as the absence of initial symptoms, the patient’s lack of knowledge about the disease, and the fear of diagnosis. Moreover, a late diagnosis can be associated with the lack of access to specialized services or diagnostic failure in primary care, exposing flaws in the health care system (26). In Brazil, although the implementation of health policies has expanded the access to oral health services and favored opportunistic screening for oral cancer, failures in early cancer detection by dental professionals are still common (27). This fact highlights the need to reinforce relevant basic knowledge about the prevention and early diagnosis of oral cancer throughout the undergraduate dental course and the adoption of continued educational strategies for professionals (28).

A single therapeutic modality was associated with lower survival rates in this study compared to the use of combination therapies. These results are similar to other studies, which found a better prognosis when adjuvant radiotherapy or chemotherapy was used after surgical treatment (20). Some authors observed that patients who were treated surgically, with or without the use of adjuvant therapy, had a much longer survival time than those not submitted to any surgical intervention (29). Since surgery is mostly indicated for smaller tumors, these findings reinforce the importance of an early diagnosis, as patients with advanced-stage inoperable tumors can only receive radio/chemotherapy as palliative treatment, worsening their prognosis (20,29).

In the current study, there was no association between survival outcomes and anatomical site of the tumor. However, lower survival rates have been related to neoplasms located at the base of the tongue and oropharynx. Locoregional metastasis was found to be more frequent in these cases due to the significant lymphatic vascularization in the region, with a consequent poorer prognosis (6). Moreover, some anatomical sites are challenging for diagnosis and treatment and can influence tumor progression and prognosis. In this respect, the lip region is more accessible, facilitating early detection and diagnosis and resulting in better survival rates (16).

No association between the presence of comorbidities and survival time was found in this study. However, the presence of comorbidities has been reported as an important factor in assessing the survival of patients with oral cancer, as the time required to investigate and monitor these other diseases can lead to delay in antineoplastic treatment (30). In addition, depending on the type and severity of the comorbidity, therapeutic modalities such as surgical resection may be contraindicated, negatively impacting patient prognosis (17). The frequency of comorbidities was considered low in the sample studied.

The main limitation of the current study is the use of secondary data, which may be associated with incomplete or incorrect medical records. However, given the robust statistical analysis, these limitations are unlikely to compromise our results. The data of this study refer to a considerable sample size and a long observation period, providing important evidence of factors associated with the prognosis of OSCC patients, which is a crucial field of study in public health.

## Conclusions

Advanced clinical stage, younger age at diagnosis, and single treatment predict poorer survival in patients diagnosed with OSCC. These findings encourage further research on factors that could modify disease outcomes in patients with OSCC.
